# Imported Fire Ants Discard Cricket Eggs

**DOI:** 10.3390/insects15120954

**Published:** 2024-11-30

**Authors:** Jian Chen, Xinzhi Ni, Michael J. Grodowitz

**Affiliations:** 1 Biological Control of Pests Research Unit, United States Department of Agriculture-Agricultural Research Service, 59 Lee Road, Stoneville, MS 38776, USA; michael.grodowitz@usda.gov; 2 Crop Genetics and Breeding Research Unit, United States Department of Agriculture-Agricultural Research Service, Tifton, GA 31793, USA; xinzhi.ni@usda.gov

**Keywords:** fatty acid, necrophoresis, chemical defense

## Abstract

The house cricket, *Acheta domesticus* (Linnaeus), is often used as a food source for the maintenance of imported fire ants under laboratory rearing. It was found that both red imported fire ants, *Solenopsis invicta* Buren, and black imported fire ants, *S. richteri* Forel, consumed most of the soft tissues of female crickets, but avoided their eggs by disposing of them on refuse piles. Bioassays using freshly collected cricket eggs showed that ants first retrieved eggs into their nests and then discarded them onto the refuse piles. The major chemicals on the surface of cricket eggs were found to be fatty acids, including lauric, myristic, palmitoleic, palmitic, linoleic, oleic, and stearic acid. Fatty acids are well-known death cues of insects and elicitors of widespread necrophoric behavior in ants. It was shown that both the cricket egg extract and the reconstructed fatty acid mixture elicited the necrophoric behavior of *S. invicta*; however, they never elicited retrieving behavior. Unknown chemicals on cricket eggs, other than fatty acids, might be responsible for the retrieving behavior. Interestingly, cricket eggs had a very similar fatty acid profile to that of dead ants collected from refuse piles. Possible causes for such a strong match in fatty acid profiles between dead ants and cricket eggs are discussed.

## Simple Summary

During routine feeding of crickets to laboratory imported fire ant colonies, it was observed that while most of the cricket tissues are used by the fire ants, only the hard outside shell or cuticle and the eggs are not utilized. Interestingly, the eggs are removed from the nest and deposited on refuse piles created by the fire ants. Why waste a highly nutritious food source, such as cricket eggs? It was found through several experimental trials and chemical analyses that the surface of the cricket egg has a chemical profile consisting mainly of fatty acids, like that found on dead fire ants. It thus elicits what is known as necrophoric behavior, where chemical cues on the surface of the ants elicit a behavior in which the dead ants are removed from the colony and deposited in refuse piles. Possible reasons for such similar surface compositions between fire ants and cricket eggs are discussed.

## 1. Introduction

Native to South America, the red imported fire ant, *Solenopsis invicta* Buren (Hymenoptera: Formicidae), has been introduced into many countries and regions and has become a global pest ant [1]. In addition to being a significant threat to public health and an important pest in agriculture, *S. invicta* also exerts a variety of ecological impacts [2]. Their impacts are so severe throughout the world that the species has been included in the 100 World’s Worst Invasive Alien Species [3]. *S. invicta* has become a dominant ant species wherever it has become established. It is a major predator of many other insects [4]. Like many other ant species, *S. invicta* has been identified as an insect oophage, i.e., an organism that consumes insect eggs. For example, it is a known predator on the eggs of several significant pest insects, including *Helicoverpa zea* (Boddie), *Spodopteran exigua* (Hübner), *Heliothis virescens* (F.), (Lepidoptera: Noctuidae) [5,6,7], *Diatraea saccharalis* (F.) (Lepidoptera: Pyralidae) [8], leaf footed bugs (Hemiptera: Coreidae) [9], and *Psorophora columbiae* (Dyar and Knab) (Diptera: Culicidae) [10].

**Figure 1:**
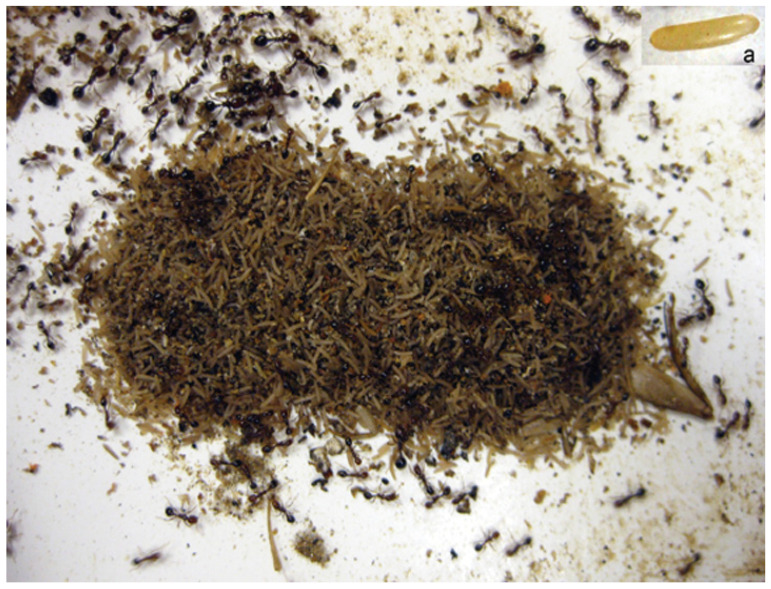
A refuse pile with house cricket eggs (an egg inset in panel **a**) that fire ant workers deposited.

House crickets, *Acheta domesticus* (Linnaeus), are often used as food sources for laboratory colonies of imported fire ants. We observed that *S. invicta* consumed most of the soft tissues of female house crickets, but often avoided their eggs by disposing them on refuse piles outside of the main nest (Figure 1), resembling the stereotypical necrophoric behavior of ants. Necrophoric behavior refers to the removal of dead colony members from the nest, an innate hygienic behavior common to many eusocial insects, including ants [11,12,13,14,15,16,17,18,19]. It is believed that the necrophoric behavior evolved under the pressure of epidemic diseases and is an essential adaptation to social life [20,21]. Ants often live in a confined nest environment that favors microbial growth; therefore, the timely removal of corpses carrying infectious disease is critical to colony health. Wilson et al. (1958) pioneered the study of chemical cues that elicit necrophoric behavior in two ant species, *Pogonomyrmex badius* (Latreille) and *Solenopsis saevissima* (Smith). Fatty acids, particularly oleic acid, were suggested as elicitors of necrophoric behavior [14]. The fatty acids on ant corpses were proposed to be the product of fat decomposition. This work is the foundation of the “fatty acid hypothesis” for explaining ant necrophoric behavior. The “fatty acid hypothesis” was supported by studies on other ant species. For example, it was found that oleic acid and other unsaturated fatty acids released necrophoric behavior in *S. invicta* [12] and oleic acid on paper bits elicited the necrophoric response in *Myrmecia vindex* Smith [22].

Despite its wide acceptance, the “fatty acid hypothesis” has been challenged due to its inability to explain some responses of ants toward fatty acids. For example, the response of *Pogonomyrmex badius* to oleic acid depends on the social context. In addition to necrophoric behavior, oleic acid also releases foraging behavior [16]. *S. invicta* expresses necrophoric behavior toward ants that were killed within one hour. It is questionable that decomposition and release of fatty acids occur in such a short time. Therefore, an alternative “mask hypothesis” has been suggested [17]. In this hypothesis, the releaser of necrophoric behavior is believed to be masked by competing odors, which gradually fade after death. However, this hypothesis has only been demonstrated in the Argentine ant *Linepithema humile* (Mayr) [23]. In this case, triglycerides were identified as the preexisting releasers of necrophoric behavior, while dolichodial and iridomyrmecin on live workers inhibited the expression of such behavior. Qian and Zhou argued that recognition of fatty acid death cues may be an evolutionary conserved response from non-eusocial ancestors to avoid the dead and that death recognition through diminished chemicals associated with life might be an evolutionary novelty in some eusocial insects [21].

Since fatty acids have long been known as elicitors of necrophoric behavior in *S. invicta*, we hypothesized that cricket eggs contain fatty acids on the egg surface. Chemical analysis was conducted not only on cricket eggs but also on dead ants. Responses of ants to newly collected cricket eggs and filter paper bits treated with the reconstructed fatty acid mixture were observed in the laboratory. The responses of ants to cricket eggs were also observed in the field.

## 2. Materials and Methods

### 2.1. Insects

This study did not involve endangered or protected species. Adult house crickets were purchased from Armstrong’s Cricket Farm in West Monroe, Louisiana. A petri dish (100 × 25 mm) filled with moist play sand was placed in each cricket bucket (34.8 × 28.5 cm). Female crickets lay eggs in the sand. Sand was collected two to three times per week, depending on the quantity of eggs produced and the dryness of the sand. No sand was placed in the buckets over the weekend. The collected sand was then spread out for drying so that it would be more easily separated from the eggs. As soon as the sand was relatively dry, a #35 sieve was used to separate the sand from the eggs. The eggs were then either individually removed from the sieve or poured into vials.

### 2.2. Fatty Acid Analysis

*Extraction and derivatization*. Fatty acids were methylated before being analyzed using gas chromatography–mass spectrometry (GC–MS). Either 50 cricket eggs or 50 dead worker ants were extracted with 1 mL hexane for 10 s in a 2 mL vial. The hexane was then transferred to a new vial. After the hexane was evaporated under an air flow, 100 µL BF_3_ of methanol (10%) was added to the vial. The vial was tightly capped and heated in a water bath at 100 °C for 20 min. After the sample cooled, 400 µL distilled water and 200 µL hexane were added into the vial. The vial was shaken vigorously 10 times. After the two layers were separated, 2 µL of hexane was injected into the GC–MS. For fatty acid quantification, 10 µL 0.32 mg/mL heptadecanoic acid was used as an internal standard. For fire ants, column chromatography was used to remove the venom alkaloids that interfere with some fatty acid methyl esters in GC–MS analysis. A disposable borosilicate glass Pasteur pipette (approx. length: 14.6 cm, Fisher Scientific, Pittsburgh, PA, USA) was used as the column. The pipette tip was first blocked using a piece of glass wool, and 0.7 g silica gel (Davisil^®^, Grade 636, pore size 60 Å, 35–60 mesh, St. Louis, MO, USA) was then filled into the pipette. The extraction after methylation was loaded onto the column. A hexane/acetone mixture was used as the mobile phase. The procedure consisted of the following two steps: 20/0, 5 mL and 17/3, 5 mL. Only the eluent of the last step was collected and used for GC–MS analysis.

*Gas chromatography–mass spectrometry.* The chemical identities of fatty acid methyl esters were achieved by matching the retention times and mass spectra of the samples with those of the standards, including lauric, myristic, palmitoleic, palmitic, linoleic, oleic, and stearic acid methyl esters. A Varian GC–MS system was used for this study. It consisted of a CP-3800 gas chromatograph and a Saturn 2000 mass selective detector, which were controlled by Mass Spectrometry WorkStation Version 6.4.1 (Varian, Walnut Creek, CA, USA). A 30 m × 0.25 mm DB-1 capillary column with 0.25 µm film thickness was used (J & W Scientific, Folsom, CA, USA). The GC temperature program was as follows: initial temperature was 50 °C, held for 1 min, gradually increased to 250 °C at a rate of 20 °C/min, and held for 40 min. The split ratio was 1:10, injection temperature was 250 °C, transfer line temperature was 200 °C. Helium was used as the carrier gas, and the flow rate was 1.0 mL/min. The mass spectrometer was operated at 70 eV in electron impact mode.

### 2.3. Bioassay

**Figure 2:**
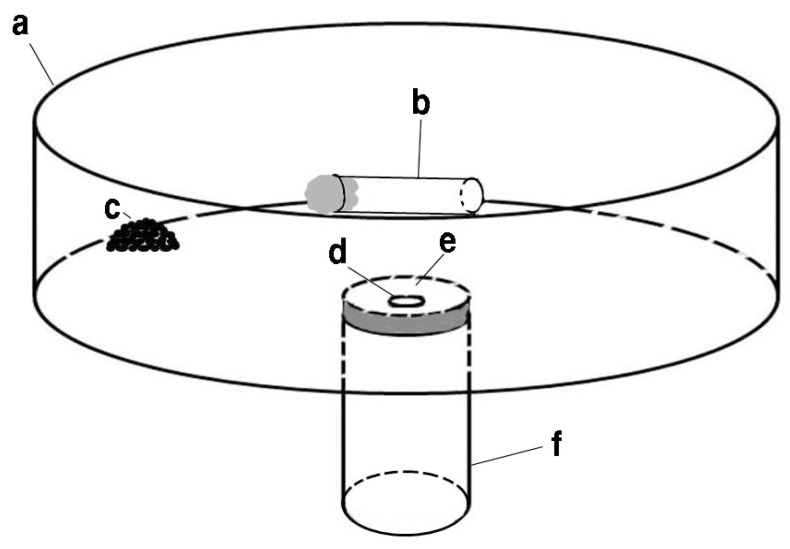
Illustration of the bioassay setup. A round plastic container (**a**) was connected to a 20 mL scintillation vial (**f**) by gluing the cap of the vial underneath the plastic container. Through a 3 mm diameter access hole (**d**), ants could reach the food source (**b**) and the refuse pile (**c**). The cricket egg or paper bit was placed in an area close to the access hole (**e**).

*Laboratory bioassay apparatus.* A plastic container (25.4 × 9 cm) provided an arena for observing ant behavior in response to cricket eggs and fatty acids (Figure 2). A 20 mL glass vial was used as an artificial nest for the ants. Underneath the plastic container, the vial (nest) was glued to the center of the plastic container. A 3 mm diameter access hole was drilled, which went through the bottom of the plastic container and the cap of the vial. The inner side of the plastic container was coated with Fluon^®^ (AGC, Tokyo, Japan). A small, reconstructed ant colony (i.e., one queen, 20 pupae, 20 larvae, and about 500 workers) was placed in the nest. Only 10% sugar water was used as a food source for the colonies during the bioassay, which was supplied in a small glass vial with the opening blocked with a cotton ball. After one or more refuse piles were formed in the arena, the colony was used for the bioassay.

*Responses to newly collected cricket eggs.* **Bioassay 1.** One egg was placed in the arena about 0.5 cm from the access hole (Figure 2, dotted line). The pick-up time (time elapsed from when an egg was placed in the arena until it was picked up by an ant) and its initial destination were recorded. Three colonies of ants were used, and 20 cricket eggs were examined for each colony. Since the data followed a normal distribution, analysis of variance (ANOVA) was used to compare differences among colonies. **Bioassay 2.** Fifty newly collected eggs were placed in the nest. The number of eggs that had been transported out of the nest was recorded every 12 h. There were six replicates, and a different colony was used for each replicate. **Bioassay 3.** In the field, cricket eggs were presented to fire ants to determine whether they would collect cricket eggs. One cricket egg was placed on the ground. The time when the ant picked up the egg was recorded. There were 53 replicates. The bioassays were conducted at 8:30 to 9:30 a.m. on 22 July 2011, on Mississippi River Levee, Washington County, Mississippi. Permission to conduct fire ant bioassays along the Mainline Mississippi River Levee in Washington County was issued by the Board of Mississippi Levee Commissioners.

*Response to cricket egg extract.* An amount of 130 mg of freshly collected cricket eggs was extracted five times in 2 mL hexane and acetone solution (1:1) for 2 min. The pooled extract (~10 mL) was used to treat 130 mg paper bits (0.345 ± 0.34 mg, mean weight ± SD). The solvent was totally evaporated before the paper bits were used in the bioassay. Paper bits treated with solvent only were used as a control. Three colonies were selected for this bioassay. For each colony, 25 control paper bits and 25 treated paper bits were tested. One paper bit was tested at a time. Control and treated paper bits were tested alternately. The paper bit was positioned at about 0.5 cm from the access hole, and the pick-up time and destination of each paper bit were recorded. The observation time for each paper bit was 30 min. Since the data did not follow a normal distribution, the Wilcoxon rank-sum test was used to compare treated and control paper bits for each colony. Data from one colony were used as a replicate when comparing percentages of paper bits transported onto refuse piles within 30 min.

*Response to reconstructed mixture of fatty acids.* Four hundred filter paper bits (0.345 ± 0.34 mg, mean weight (mg) ± SD) were washed three times using 1.0 mL acetone. Of these, 200 paper bits were treated with fatty acid solution. The relative abundance of each fatty acid in the mixture was 0.31% lauric acid, 0.67% myristic acid, 1.56% palmitoleic acid, 26.71% palmitic acid, 29.68% linoleic acid, 32.33% oleic acid, and 8.73% stearic acid. The final concentration of total fatty acid on the paper bits was 10.91 µg/mg, which was equivalent to the amount of fatty acids on the cricket eggs. For each of the three colonies, 25 control paper bits and 25 treated paper bits were tested. One paper bit was tested at a time. Control and treated paper bits were tested alternately. The paper bit was positioned about 0.5 cm from the access hole, and the pick-up time and destination of each paper bit were recorded. The observation time for each paper bit was 30 min. Since the data did not follow a normal distribution, the Wilcoxon rank-sum test was used to compare treated and control paper bits for each colony. Data from one colony were used as a replicate when comparing percentages of paper bits transported onto refuse piles within 30 min.

## 3. Results

**Figure 3:**
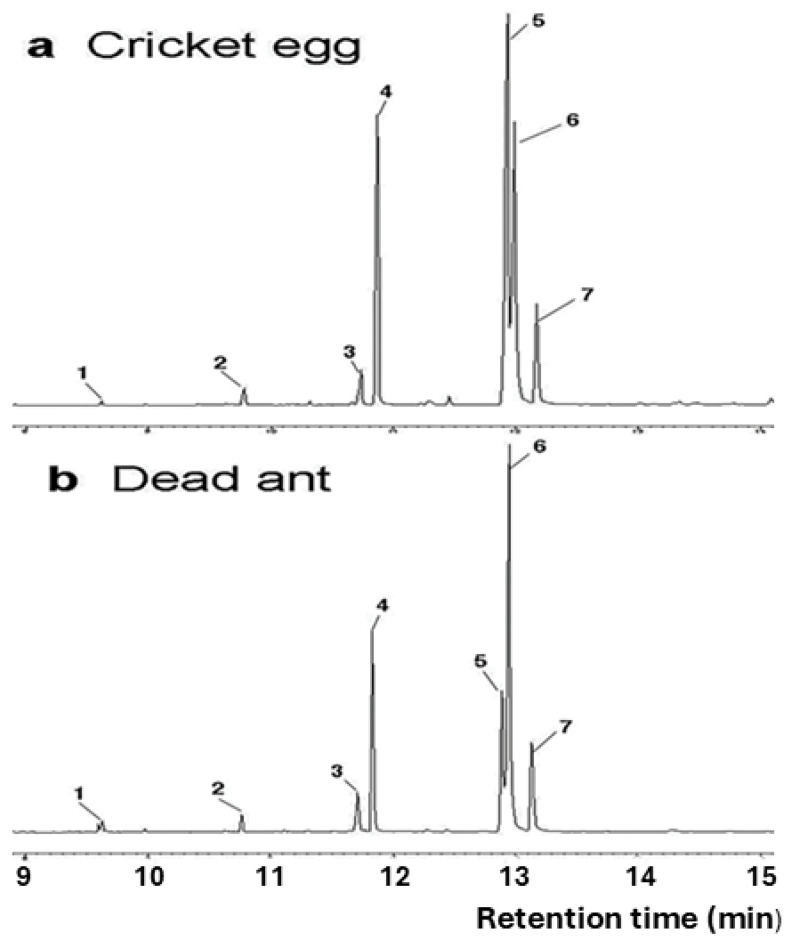
Chromatograms of fatty acids found on house cricket eggs (**a**), dead workers of the red imported fire ants (**b**). Fatty acids were methylated using BF_3_ in methanol. Peak assignment: 1. lauric acid methyl ester, 2. Myristic acid methyl ester, 3. palmitoleic acid methyl ester, 4. palmitic acid methyl ester, 5. linoleic acid methyl ester, 6. oleic acid methyl ester, 7. stearic acid methyl ester.

*Fatty acids on cricket eggs and dead ants.* Chemical analysis of house cricket eggs revealed that fatty acids are major components on the surface of cricket eggs (Figure 3a). The fatty acid profile of the cricket eggs is highly like that of dead fire ants collected from a refuse pile (Figure 3b). The following seven fatty acids were found on house cricket eggs and dead fire ants: lauric, myristic, palmitoleic, palmitic, linoleic, oleic, and stearic acids. Qualitatively, there was a strong match between the fatty acid profiles of house cricket eggs and dead fire ants. Relative content (%) and absolute amount of individual fatty acid on cricket eggs are shown in Table 1. Palmitic, linoleic, and oleic acids were always the most abundant fatty acids on both cricket eggs and dead ants. The total amount of fatty acids on a cricket egg was 3.96 ± 1.33 µg/egg (mean ± SE). Based on the fatty acid percentage data, cosine similarity and Pearson correlation were calculated. Both cosine similarity (0.959) and Pearson correlation (0.871) were high, suggesting strong proportional and trend-based similarities.

**Figure 4:**
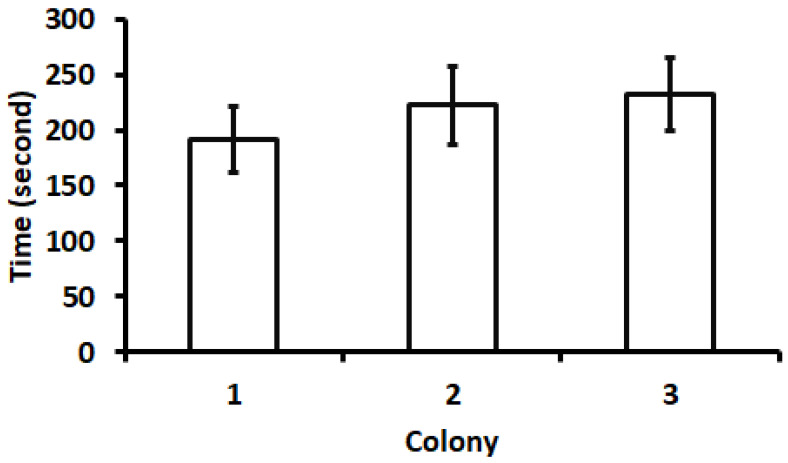
Mean time elapsed from when an egg was placed in the arena until it was picked up by an ant (pick-up time). There were no significant differences in mean pick-up time among the three colonies (*F* = 0.41; *df* = 2, 57; *p* = 0.67).

**Figure 5:**
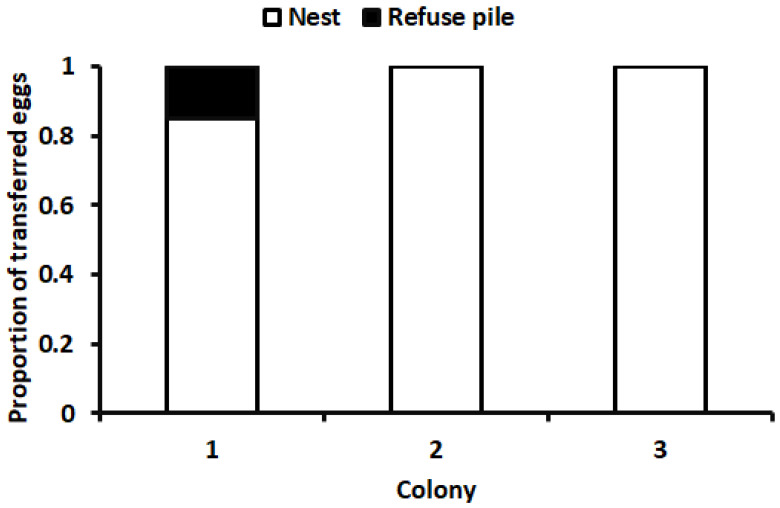
Proportion of cricket eggs initially transported to nest or refuse piles.

**Figure 6:**
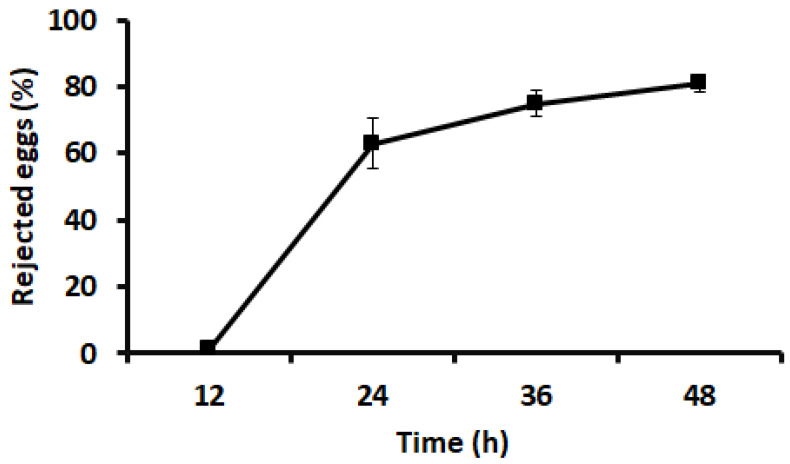
Percentage of cricket eggs rejected by ants as a function of time.

*Responses to newly collected cricket eggs.* For the first bioassay, the mean pick-up time (time elapsed from when a cricket egg was placed in the arena until it was picked up by an ant) is shown in Figure 4. The minimum pick-up time was 5 s, and the maximum was 536 s. There were no significant differences in the pick-up times among the three colonies (*F* = 0.41; *df* = 2, 57; *p* = 0.67). In two of the three colonies, the ants transported all the eggs into the nest; in the other colony, the ants transported 17 of the 20 eggs into the nest and the remaining three eggs directly to refuse piles (Figure 5). In bioassay 2, after newly collected eggs were placed in the nest, 81% of the eggs were transferred out of the nest within 48 h (Figure 6). In the third bioassay on the response of ants to cricket eggs in the field, eggs were collected by ants in all 53 cases. The pick-up time was 160.91 ± 22.03 s (mean ± SE). The minimum pick-up time was 13 s and the maximum was 930 s.

**Figure 7:**
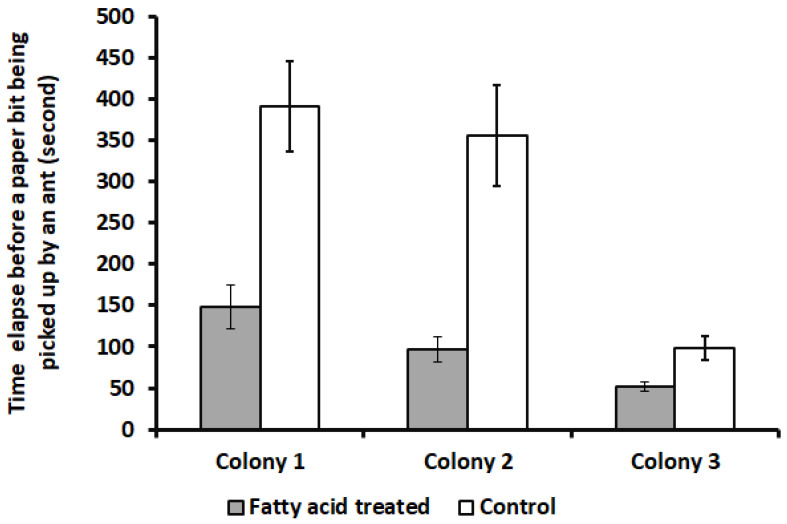
Mean time elapsed from when a paper bit was placed in the arena until it was picked up by an ant (pick-up time). Paper bits treated with fatty acid mixture had significantly smaller pick-up times than the control paper bits for each colony (Wilcoxon rank-sum test. Colony 1: z = 3.65, *p* = 0.0003; Colony 2: z = 4.33, *p* < 0.0001; Colony 3: z = 2.98, *p* = 0.001).

**Figure 8:**
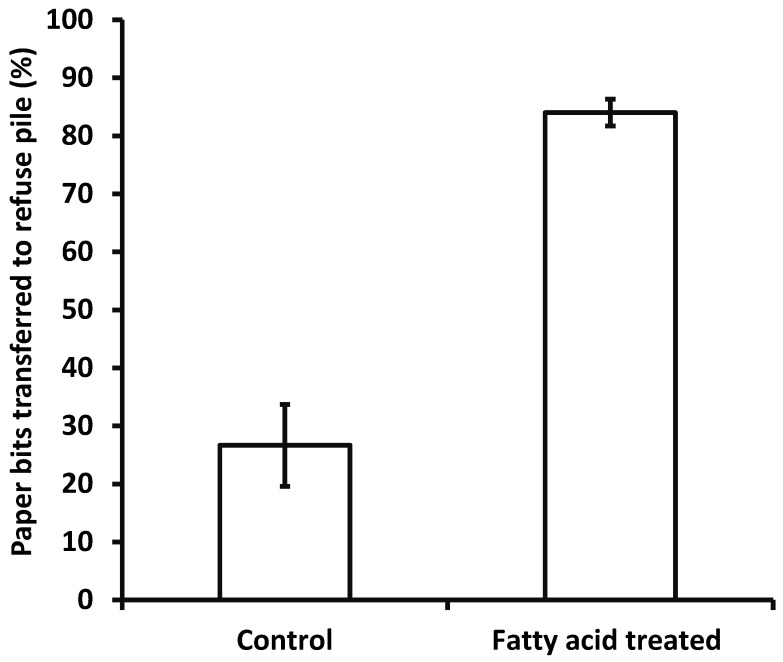
Percentage of paper bits transported into refuse piles within 30 min after being placed in the arena (*N* = 25 for each of 3 colonies). Data from one colony were used as a replicate. Significantly more treated paper bits were transported into the refuse piles within 30 min (means with different letters are significantly different. Wilcoxon rank-sum test, z = 1.75, *p* = 0.04).

*Response to reconstructed mixture of fatty acids.* Fatty acid mixture significantly reduced the pick-up time (Figure 7) (Wilcoxon rank-sum test, colony 1: z = 3.65, *p* = 0.0003; colony 2: z = 4.33, *p* < 0.0001; colony 3: z = 2.98, *p* = 0.001) and significantly increased the proportion of paper bits that were transferred onto refuse piles within 30 min (Figure 8) (Wilcoxon rank-sum test, z = 1.75, *p* = 0.04).

## 4. Discussion

The utilization of fatty acids in the chemical defense of insect eggs has been reported. For example, oleic acid in the eggs of the chrysomelid *Gastrophysa cyanea* (Melsheimer, 1847) (Coleoptera: Chrysomelidae) serves as an effective feeding deterrent for ants [14]. A single egg of *G. cyanea* contains about 40 µg oleic acid. The egg stalk of the chrysopid *Ceraechrysa smithi* (Návas) (Neuroptera: Chrysopidae) is coated in a protective secretion that repels ants [15], and oleic and linoleic acids are major components of that secretion. Palmitoleic, linoleic, and oleic acids in the sternal gland secretion of adult females of *Polistes dominula* (Christ.) and *P. sulcifer* Zimmermann (Hymenoptera, Vespidae) have a repellant effect on many ant species [16]. In a study of the chemical defenses of two nymphalid butterfly larvae against *Camponotus rufipes* Forel (Hymenoptera: Formicidae), linoleic and oleic acids were also found to deter ant attacks [17]. It was found that a combination of linoleic and oleic acid was more effective than individual acids, showing a potential synergism among fatty acids in deterring ants [17]. However, what was observed in this study is much more complicated. In most cases, cricket eggs were initially retrieved into the ant nest before they were discarded onto refuse piles. In a previous study with *Pogonomyrmex badius* (Latreille) (Hymenoptera: Formicidae)*,* oleic acid was found to elicit two seemingly opposing behaviors of ants, foraging and necrophoric, which depended on the social context, i.e., the nest activities at that particular moment [16]. Ants in different modes of task allocation may have different responses to certain chemicals. It is most likely that the eggs were first collected by foragers and, once they were in the nest, were then discarded by ants that were maintaining the hygiene of the colony. However, since fire ants did not retrieve a single paper bit treated with fatty acid mixture, fatty acids may not be the only reason for the initial retrieving behavior observed in this study.

It was reported that ants collected and then discarded the eggs of some walkingsticks [24,25]. This is probably the most similar case to that observed in this study. However, one significant difference between crickets and walkingsticks is that the egg of the walkingstick is hard-shelled and has a capitulum, i.e., a detachable extension of the opercular plate. It is believed that capitulum causes ants to transport walkingstick eggs into their nests. Ants remove and consume the capitulum within their nests and eventually discard the eggs. It is the hard shell that prevents the walkingstick eggs from being consumed by ants in the nest. Ants help walkingsticks disperse their progeny. In contrast, the cricket egg is soft-shelled and does not have a capitulum. Cricket eggs could easily be consumed by ants in the nest if chemical defenses were not present.

There are two possible explanations for the strong match in fatty acid profiles between dead ants and cricket eggs: (1) a random coincidence, and (2) a chemical mimicry that is an evolutionary consequence of the crickets driven by predation pressure of ants. Living in the soil, cricket eggs must be exposed to various selection pressures. One obvious pressure is from pathogenic microorganisms. The antimicrobial activities of free fatty acids are well documented. They have shown diverse biological activities, including anti-algal, antibacterial, antifungal, antiprotozoal, and antiviral [26]. For example, fatty acids are key antimicrobial ingredients in food additives [27] and antibacterial herbs [28,29]. It is possible that the evolution of the chemical protection of cricket eggs using fatty acids has been driven by pathogenic microorganisms in the soil. The strong match of fatty acid profiles between cricket eggs and dead ants may be a pure evolutionary coincidence.

Since house crickets are most likely native to Southwestern Asia, they might not have an opportunity for coevolution with the red imported fire ants. However, since fatty acids are universal death cues [30], avoidance of corpses by responding to fatty acids may be an evolutionary conserved trait for animals in general. Mimicking death cues to avoid predation has a great advantage because such mimicry may target a broad range of enemies simultaneously. In a study on the impact of polygyne *S. invicta* on surface-active arthropods at a field station in central Texas, it was found that *S. invicta* had a significant impact on the indigenous insect fauna. However, in contrast to a significant decline in the abundance of isopods, erythraeid mites, and tumblebug scarabs, the abundance of ground crickets increased significantly. Unfortunately, the underlying causes of such increased abundance have not been investigated. It will be interesting to see whether the eggs of ground crickets also have fatty acid profiles similar to those of dead fire ant ants. The interaction between ground crickets and fire ants may provide a good opportunity to further test the fatty acid mimicry hypothesis.

## Figures and Tables

**Table 1:** Relative content (%) and absolute concentration of individual fatty acids on cricket eggs and dead ants.

Insect	Relative content of fatty acid (%, mean ± SE)				
	palmitoleic acid	palmitic acid	linoleic acid	oleic acid	stearic acid
Cricket eggs	2.13 ± 0.48	25.39 ± 0.89	26.46 ± 0.78	39.97 ± 0.93	6.05 ± 0.55
Dead ants	7.42 ± 0.62	17.40 ± 1.28	17.75 ± 0.94	44.01 ± 1.70	13.43 ± 0.98
	**Absolute concentration of fatty acid (µg/egg, mean ± SE)**				
	**palmitoleic acid**	**palmitic acid**	**linoleic acid**	**oleic acid**	**stearic acid**
Cricket eggs	0.089 ± 0.035	1.002 ± 0.347	1.031 ± 0.338	1.580 ± 0.522	0.254 ± 0.098
Dead ants	0.239 ± 0.099	0.861 ± 0.209	1.283 ± 0.380	1.784 ± 0.467	0.298 ± 0.132

## Data Availability

The raw data supporting the conclusions of this article will be made available by the authors on request.

## References

[1] Ascunce M.S., Yang C.C., Oakey J., Calcaterra L., Wu W.J., Shih C.J., Goudet J., Ross K.G., Shoemaker D. (2011). Global invasion history of the fire ant Solenopsis invicta. Science.

[2] Vinson S.B. (2013). Impact of the invasion of the imported fire ant. Insect Sci..

[3] Lowe S., Browne M., Boudjelas S., De Poorter M. (2000). 100 of the World’s Worst Invasive Alien Species: A Selection from the Global Invasive Species Database.

[4] Hölldobler B., Wilson E.O. (1990). The Ants.

[5] Pfannenstiel R.S., Hoddle M.E. (2005). Nocturnal predators and their impact lepidopteran eggs in annual crops: What we don’t see does help us!. Proceedings of the Second International Symposium on Biological Control of Arthropods.

[6] Nuessly G.S., Sterling W.L. (1994). Mortality of Helicoverpa zea (Lepidoptera: Noctuidae) eggs in cotton as a function of oviposition sites, predator species, and desiccation. Environ. Entomol..

[7] McDaniel S.G., Sterling W.L. (1982). Predation of *Heliothis virescens* (F.) eggs on cotton in East Texas. Environ. Entomol..

[8] Negm A.A., Hensley S.D. (1969). Evaluation of certain biological control agents of the sugarcane borer in Louisiana. J. Econ. Entomol..

[9] Mitchell P.L., Paysen E.S., Muckenfuss A.E., Schaffer M., Shepard B.M. (1999). Natural mortality of leaffooted bug (Hemiptera: Heteroptera: Coreidae) eggs in cowpea. J. Agric. Urban Entomol..

[10] Lee D.K. (1989). The Effects of Certain Physical and Biological Factors on the Survival and the Hatching Rates of Psorophora Columbiae (Dyar and Knab) (Diptera: Culicidae) Eggs. Ph.D. Thesis.

[11] Diez L., Moquet L., Detrain C. (2013). Post-mortem changes in chemical profile and their influence on corpse removal in ants. J. Chem. Ecol..

[12] Blum M.S., Beroza M. (1970). The chemical basis of insect sociality. Chemicals Controlling Insect Behavior.

[13] Oi D.H., Pereira R.M. (1993). Ant Behavior and Microbial Pathogens (Hymenoptera: Formicidae). Fla. Entomol..

[14] Wilson E.O., Durlach N.I., Roth L.M. (1958). Chemical releasers of necrophoric behavior in ants. Psyche.

[15] Haskins C.P., Haskins E.F. (1974). Notes on the necrophoric behaviour in the archaic ant Myrmecia vindex. Psyche.

[16] Gordon D.M. (1983). Dependence of necrophoric response to oleic acid on social context in the ant, Pogonomyrmex badius. J. Chem. Ecol..

[17] Howard D.F., Tschinkel W.R. (1976). Aspects of necrophoric behavior in the red imported fire ant. Solenopsis invicta. Behaviour.

[18] Renucci M., Tirard A., Provost E. (2011). Complex undertaking behavior in *Temnothorax lichtensteini* ant colonies: From corpse-burying behavior to necrophoric behavior. Insectes Sociaux.

[19] Choe D.H. (2009). Necrophoric Behavior of the Argentine Ant, Linepithema Humile (Mayr) (Hymenoptera: Formicidae), and Its Implications for Horizontal Transfer of Slow-Acting iNsecticides. Ph.D. Thesis.

[20] Fefferman N.H., Traniello J.F.A., Rosengaus R.B., Calleri I.D.V. (2007). Disease prevention and resistance in social insects: Modeling the survival consequences of immunity, hygienic behavior, and colony organization. Behav. Ecol. Sociobiol..

[21] Sun Q., Zhou X. (2013). Corpse management in social insects. Int. J. Biol. Sci..

[22] Haskins C.P., Aronson L.R., Tobach E., Lehrman D.S., Rosenblatt J.S. (1970). Researches in the biology and social behavior of primitive ants. Development and Evolution of Behavior.

[23] Choe D.H., Millar J.G., Rust M.K. (2009). Chemical signals associated with life inhibit necrophoresis in Argentine ants. Proc. Natl. Acad. Sci. USA.

[24] Compton S.G., Ware A.B. (1991). Ants disperse the elaiosome-bearing eggs of an African stick insect. Psyche.

[25] Hughes L., Westoby M. (1992). Capitula on stick insect eggs and elaiosomes on seeds: Convergent adaptations for burial by ants. Funct. Ecol..

[26] Desbois A.P., Smith V.J. (2010). Antibacterial free fatty acids: Activities, mechanisms of action and biotechnological potential. Appl. Microbiol. Biotechnol..

[27] Freese E., Shew C.W., Galliers E. (1973). Function of lipophilic acids as antimicrobial food additives. Nature.

[28] Dilika F., Bremner P.D., Meyer J.J.M. (2000). Antibacterial activity of linoleic and oleic acids isolated from *Helichrysum pedunculatum*: A plant used during circumcision rites. Fitoterapia.

[29] McGaw L.J., Jäger A.K., van Staden J. (2002). Isolation of antibacterial fatty acids from *Schotia brachypetala*. Fitoterapia.

[30] Yao M., Rosenfeld J., Attridge S., Sidhu S., Aksenov V., Rollo C.D. (2009). The ancient chemistry of avoiding risks of predation and disease. Evol. Biol..

